# Co-application of leguminous and non-leguminous green manures enhances subsequent wheat yield stability in saline-alkali soils

**DOI:** 10.3389/fpls.2026.1850670

**Published:** 2026-06-17

**Authors:** Mengxuan Zhang, Ru Yu, Jufeng Cao, Weiqian Li, Shaogang Tang, Zhuangzhuang Chen, Hanjiang Liu, Xuemin Wang, Jinjun Cai, Hongyuan Zhang

**Affiliations:** 1 State Key Laboratory of Efficient Utilization of Arable Land in China, The Institute of Agricultural Resources and Regional Planning, Chinese Academy of Agricultural Sciences, Beijing, China; 2 School of Ecology and Biology, Dongying Vocational College, Dongying, China; 3 Bayannur Academy of Agricultural and Animal Husbandry Sciences, Linhe, China; 4 Institute of Agricultural Resources and Environment, Ningxia Academy of Agriculture and Forestry Sciences, Yinchuan, China; 5 Crop Research Institute, Ningxia Academy of Agriculture and Forestry Sciences, Yinchuan, China; 6 National Center of Technology Innovation for Comprehensive Utilization of Saline-Alkali Land, Dongying, China

**Keywords:** green manure, nitrogen fertilizer, saline-alkali soils, soil ecosystem multifunctionality, spring wheat yield

## Abstract

The incorporation of green manure into cropping systems is recognized as an effective practice for improving environmental sustainability, particularly in irrigated agroecosystems such as the Hetao Irrigation District. However, the effect of green manure as a preceding crop in regulating subsequent wheat productivity and soil functioning in saline soils under limited nitrogen (N) inputs remains insufficiently understood. To address this gap, a 9-year field experiment was conducted to evaluate the effects of green manure types (non-leguminous feed rape (GMR), leguminous hairy vetch (GMV), and their combined application (GMRV) and N application rates (N150, 150 kg N per ha; N0, no N input) on multi-year spring wheat yield, soil ecosystem multifunctionality (EMF), and microbial resource limitation. The results showed that only green manure types had significant influence on subsequent yield and stability except for year. Compared with GMR, the GMRV treatment increased average yield by 17.2% under N150 and enhanced yield stability by 21.7% and 22.2% under N150 and N0, respectively. In addition, GMRV markedly enhanced soil organic carbon (SOC), enzyme activities, and EMF across both the wheat-growing and green manure seasons. Positive relationships were observed among SOC, total nitrogen (TN), EMF, and crop yield. Microbial communities were primarily constrained by C and N availability. GMRV reduced the microbial C limitation during the green manure season, while GMV alleviated the microbial C limitation at spring wheat season. In conclusion, the combined application of leguminous and non-leguminous green manure improved crop productivity and soil functioning by promoting SOC accumulation, nutrient availability, and ecosystem multifunctionality, while alleviating microbial resource constraints. This approach offers a promising pathway for reducing nitrogen fertilizer dependence in saline agroecosystems such as the Hetao Irrigation District.

## 1 Introduction

Addressing the growing global demand for food while mitigating environmental degradation is essential for sustainable agricultural development (Li et al., 2018; Chang et al., 2023). Over the past four decades, intensive agricultural production in China has relied heavily on nitrogen fertilizers, with application rates far exceeding crop demand, leading to issues including soil degradation (Yin et al., 2021; Chang et al., 2023). Accordingly, the Chinese government has introduced a range of policy measures, such as the “Zero Growth of Chemical Fertilizer Use by 2020” initiative and the subsequent “Green Agricultural Development” strategy (Gao et al., 2023). For example, N fertilizer reduction, organic fertilizer substitution, straw return, and rotation with green manure have become effective strategies. These strategies aim to sustain or enhance crop productivity while decreasing reliance on fertilizers application, thereby contributing to national food security and sustainable agricultural development (Fischer et al., 2014; Chang et al., 2023).

The Hetao Irrigation District is a key grain-producing region in northern China. However, to sustain high crop productivity, agricultural production in this area has long been characterized by the overuse of chemical fertilizers. This has resulted in severe soil ecosystem degradation, threatening the long-term stability of spring wheat production (Yin et al., 2021). In addition, soils in the Hetao Irrigation District are widely affected by salinization and alkalization because of long-term irrigation, high evaporation, and shallow groundwater levels. These saline-alkaline soils are typically characterized by high salt accumulation, elevated pH, and low nutrient availability, which adversely affect soil fertility, nutrient cycling, and crop productivity (Haj-Amor et al., 2022). Consequently, maintaining stable wheat production under saline-alkaline conditions has become a major challenge for sustainable agricultural development in this region. Green manure incorporation has been recognized as an effective practice for improving saline-alkaline soils through increasing organic matter input and enhancing soil fertility. Under the framework of N fertilizer reduction, achieving a synergy between increasing crop yields and promoting sustainable agriculture has emerged as a central research priority. Green manure incorporation has received extensive attention owing to its cost-effectiveness, sustained nutrient release, and eco-friendly nature (Fan et al., 2026; Wang et al., 2026). Consequently, cultivating green manure during the autumn fallow period after the spring wheat harvest in this region makes full use of available light and temperature conditions while scavenging residual soil nitrogen and reducing nutrient leaching (Wang et al., 2018; Xu et al., 2024). Furthermore, the incorporation of green manure significantly increases the soil organic carbon stocks, thereby decreasing the demand for chemical fertilizers in subsequent crops (Li et al., 2024a). In the Hetao Irrigation District, it is essential to develop green manure management strategies that maintain or increase wheat yields. Increasing biodiversity within cropping systems promotes ecosystem services, which in turn support stable crop production while lowering dependence on external inputs (German et al., 2017; Tao et al., 2024). Previous studies have shown that incorporating of leguminous and non-leguminous green manures significantly improves soil organic carbon and nitrogen levels (Lee et al., 2010).

However, the effects on crop yield remain variable depending largely on the green manure type and the experimental year (Ma et al., 2021). A global meta-analysis has revealed that incorporating legumes into cropping systems results in approximately a 20% increase in yield of main crops relative to non-legume systems (Zhao et al., 2022). Compared with monoculture returning, the rice yield of Chinese milk vetch and feed rape mixed returning increased by 1.78%-7.26% (Li et al., 2010). Meanwhile, the use of green manure as a partial substitute for inorganic fertilizers can effectively sustain grain yield (Zhao et al., 2015; Urruty et al., 2016). The incorporation of leguminous green manure has a positive impact on both yield stability and agricultural sustainability (Liu et al., 2023). Although substantial research has examined the benefits of green manure on crop yields, limited attention has been given to the impacts of long-term green manure incorporation on subsequent spring wheat yield and its stability. Under saline-alkaline conditions, these yield responses are closely associated with changes in soil biological processes and nutrient cycling functions, which are fundamentally regulated by soil microbial activity and ecosystem functioning.

Recently, soil ecosystem multifunctionality (EMF) and enzymatic stoichiometry have been widely used to evaluate multiple ecosystem functions (Li et al., 2024b) and to characterize microbial resource availability and constraints (Song et al., 2023). The long-term stability of crop production is closely linked to soil nutrient cycling, a process driven by soil ecosystem multifunctionality (EMF) and microbial activity. In this context, soil enzymes play a critical role in connecting microbial metabolic demand with nutrient supply (Ye et al., 2022; Zhang et al., 2025; Khoiri et al., 2025). Therefore, enzyme activities and stoichiometry are key metrics for understanding shifts in EMF and microbial resource limitations. Previous studies have shown that agricultural management practices affect EMF (Yu et al., 2024) and microbial resource limitations by modifying soil extracellular enzyme activities (Zheng et al., 2020; Su et al., 2022). For instance, exogenous nitrogen inputs disrupt the inherent substrate balance in soils, exacerbating microbial carbon limitation (Wang et al., 2022; Kopáček et al., 2013). Conversely, green manure incorporation not only supplies abundant carbon sources and nutrient substrates for soil microorganisms but also significantly stimulates extracellular enzyme activities, thereby enhancing EMF (Cheng et al., 2025; Jia et al., 2025). Furthermore, enzyme-mediated nutrient release processes regulate the balance of microbial demand for key elements, ultimately influencing microbial resource limitation (Nevins et al., 2021). Notably, leguminous green manure has the potential to alleviate microbial carbon and nitrogen constraints via biological N fixation (Su et al., 2022). Nevertheless, our current understanding of how EMF and microbial resource limitation respond to different green manure types (leguminous and non-leguminous) in wheat–green manure rotation systems remain incomplete.

Accordingly, a long-term field experiment was established in the Hetao Irrigation District. This study aimed to evaluate (i) the effects of different green manure types and nitrogen application levels on subsequent wheat yield and its stability, and (ii) their effects on soil ecosystem multifunctionality (EMF) and microbial resource constraints. We hypothesized that (i) under reduced nitrogen inputs in green manure season, the combined incorporation of leguminous and non-leguminous green manures would significantly increase the multi-year average yield of subsequent spring wheat and enhance its interannual yield stability; and (ii) this combined incorporation would further promote SOC accumulation and enzyme activities, enhance soil ecosystem multifunctionality, and alleviate microbial resource constraints.

## 2 Materials and methods

### 2.1 Site description

**Figure 1:**
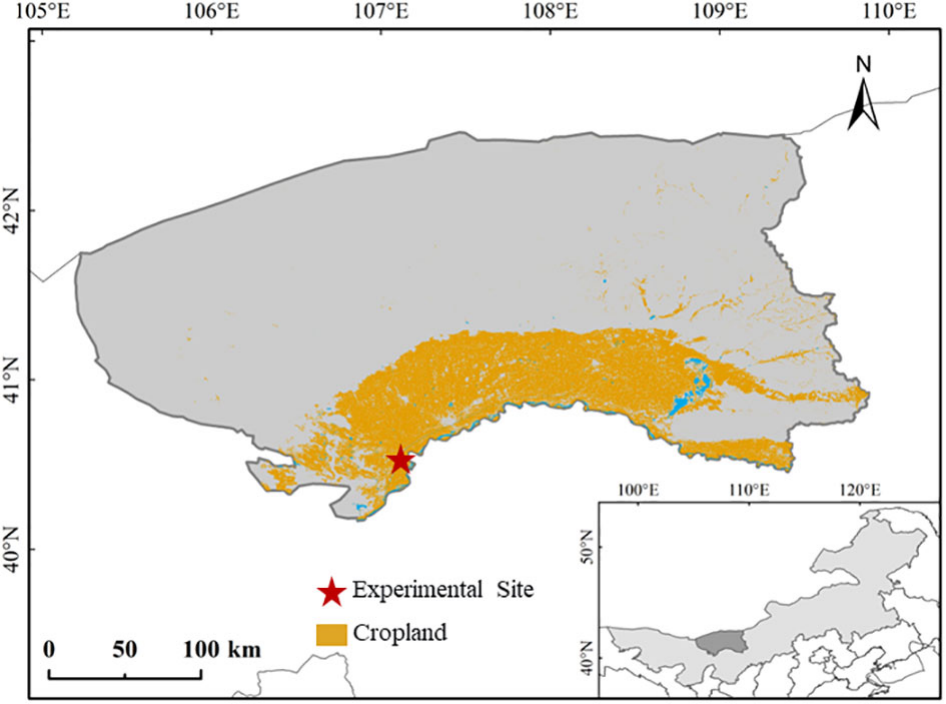
Map of the experimental site location.

The experiment began in 2015 and was set up at the Yuanzi Drainage Experiment Station (40°54’ N, 107°10’ E), Bayannaoer City, China (**Figure 1**). The field site has a mean annual temperature ranging from 3.7 °C to7.6 °C and annual precipitation of 188 mm. The field had slightly saline-alkali soil (total salt<2.0 g kg^-1^ soil). At the start of the experiment in 2016, topsoil layer (0–20 cm) was slightly saline–alkaline, with soil organic carbon (SOC), total nitrogen (TN), available phosphorus (AP), and available potassium (AK) of 9.1, 0.91, 32.5, and 140 mg kg^-1^, respectively, and a pH (H_2_O) of 8.7.

### 2.2 Experimental design

The treatments were arranged in a split-plot experimental design with three replicates, using plots measuring 4 m × 7.5 m. The main plots consisted of two N fertilization application rates: the conventional N rate used by local farmers (150 kg N ha^-1^, N150) and no N fertilizer (N0). The subplots consisted of three green manure types: non-leguminous feed rape green manure (GMR), leguminous hairy vetch (GMV), and the combined application of non-leguminous and leguminous green manure (GMRV). During the green manure growing season, fertilizers were applied as follows: base fertilizers were N of 74 kg ha^-1^, topdressing is N of 76 kg ha^-1^ at bolting stage. Specific fertilization details of green manure growing season are shown in **Supplementary Table 1**. The seeding rates of non-leguminous green manure feed rape variety (Huayouza 62), the leguminous green manure hairy vetch variety (Turkmen Vicia villosa) and their mixed sowing were 7.5, 75, and (4.5 + 60) kg ha^-1^, respectively, based on local conventional seeding practices. At the green manure full-bloom stage, the aboveground biomass was mechanically chopped into 2–3 cm segments and incorporated into the 0–25 cm soil layer using a rotary tillage system. Irrigation during the green manure season was applied twice: immediately after sowing (180 m^3^ ha^-1^) and at the branching stage (290 m^3^ ha^-1^). The subsequent crop was spring wheat (Yongliang 4), planted in March at a seeding rate of 375 kg ha^-1^. The N fertilizer application rate for subsequent spring wheat was kept consistent across all treatments, following the local conventional practice: basal N at 54 kg ha^-1^ and topdressing N at 173 kg ha^-1^. Spring wheat was irrigated four times: pre-sowing (290 m^3^ ha^-1^), jointing stage (150 m^3^ ha^-1^), heading stage (150 m^3^ ha^-1^), and grain filling stage (150 m^3^ ha^-1^). Pest control, and other management measures are consistent with normal local field production for both green manure and wheat seasons.

### 2.3 Soil sampling and determination

#### 2.3.1 Soil pH, SC, SOC, TN and TP

Soil samples were collected from the topsoil (0–20 cm) using a five-point composite sampling method within each plot in mid-July 2023 during the wheat-growing season and in early October during the green manure season. Gravel and crop residues were removed from the samples, and each soil sample was divided into two portions. One portion was air-dried and used for the determination of pH, salt content (SC), soil organic carbon (SOC), total nitrogen (TN), and total phosphorus (TP). The other subsample was immediately stored at 4 °C for the analysis of soil enzyme activities. Soil SC and pH were determined in a 1:5 (w/v) soil–water suspension prepared with deionized water. SOC and TN contents were quantified using a Vario TOC analyzer (Elementar, Germany). TP was analyzed by the molybdenum–antimony colorimetric method (Olsen and Sommers, 1982).

#### 2.3.2 Enzyme activities

The enzyme activities β-1, 4-glucosidase (BG), cellobiohydrolase (CE), xylosidase (BX), β-1,4-N-acetylglucosaminidase (NAG), leucine aminopeptidase (LAP), and phosphatase (ALP) were identified using fluorogenic substrates based on 4-methylumbelliferone (MUF) and 7-amino-4-methylcoumarin (AMC) (Jia et al., 2022). These six enzyme activities were integrated to quantify soil ecosystem multifunctionality (EMF). Specifically, individual enzyme activities were standardized using Z-score transformation (Equation 1), and the resulting values were averaged to derive a multifunctionality index (Equation 2) following Byrnes et al. (2014).

(1) $$Z-score=\frac{x-\mu_{i}}{\sigma_{i}}$$

(2) $$EMF=\frac{\sum_{1}^{n}\left(Z-score\right)}{n}$$

where *x* denotes the measured enzyme activity, *μ_i_* represents the mean value of enzyme *i*, and σ*_i_* indicates the standard deviation of enzyme *i*.

Two approaches were adopted to evaluate microbial resource limitations (Cui et al., 2022). The first method used (LAP + NAG)/AP as the x-axis and BG/(LAP + NAG) as the y-axis to construct enzyme stoichiometric scatter plots, in which different regions correspond to distinct resource limitations (Chen et al., 2019; Zheng et al., 2020). The second approach quantified vector length and angle as indicators of microbial resource limitation. Specifically, carbon (C) limitation increased with increasing vector length, while vector angles< 45° indicate N limitation and > 45° indicate P limitation (Moorhead et al., 2013). Vector length and angle were computed using Equation 3 and Equation 4 following Moorhead et al. (2016) as follows:

(3) $$Vector\;length=\sqrt{{\left[\frac{\ln\left(\text{BG}+\text{BX}+\text{CE}\right)}{\ln\left(\text{LAP}+\text{NAG}\right)}\right]}^{2}+{\left[\frac{\ln\left(\text{BG}+\text{BX}+\text{CE}\right)}{\ln\;\text{AP}}\right]}^{2}}$$

(4) $$Vector\;angle=Degress\left\{ATAN2\left[\frac{\ln\left(\text{BG}+\text{BX}+\text{CE}\right)}{\ln\;\text{AP}}\right],\left[\frac{\ln\left(\text{BG}+\text{BX}+\text{CE}\right)}{\ln\left(\text{LAP}+\text{NAG}\right)}\right]\right\}$$

#### 2.3.3 Spring wheat yield

Spring wheat yield was used as an indicator to assess the legacy effects of pre-crop green manure. At harvest, three representative quadrats, each with a circular area of 3.14 m^2^ (2 m in diameter), were randomly selected within each plot. Wheat yield was measured by threshing harvested plants after oven-drying to constant weight. Spike length, spike number and grain number were quantified by counting spikes from 10 randomly selected plants per plot prior to harvest. The 1000-grain weight was determined by weighing 1000 seeds, with 3 replicates measurements. Spikelet sterility is the ratio of sterile spikelet number to spikelet number. To evaluate yield stability of spring wheat, the coefficient of variation (CV) was computed, with lower CV indicating greater yield stability. In addition, the sustainable yield index (SYI) was applied to assess yield sustainability, where lower SYI indicates reduced yield potential. CV and SYI were calculated according to Equations 5 and 6 as follows:

(5) $$\text{CV}=\frac{\sigma }{Y_{Av}}$$

(6) $$\text{SYI}=\frac{Y_{Av}-\sigma }{Y_{max}}$$

where σ, *Y_Av_*, and *Y_max_* is the standard deviation of yield, average yield and maximum yield during the experimental years, respectively.

### 2.4 Statistical analysis

Data processing and compilation were performed using Microsoft Excel 2020. All statistical analyses were conducted using DPS software (version 9.01). A two-way analysis of variance (ANOVA) was applied to evaluate the main and interactive effects N fertilization management and green manure type on soil physicochemical properties, enzyme activities, EMF, microbial resource limitations (vector length and angle), and spring wheat yield and its components. To account for inter-annual variations, year was included as a third fixed factor in a three-way ANOVA. When significant main effects or interactions were observed, treatment means were compared using Fisher’s least significant difference (LSD) test at the 0.05 significance level. Data visualization was performed using Origin 2021.

## 3 Results

### 3.1 Spring wheat yield

**Figure 2:**
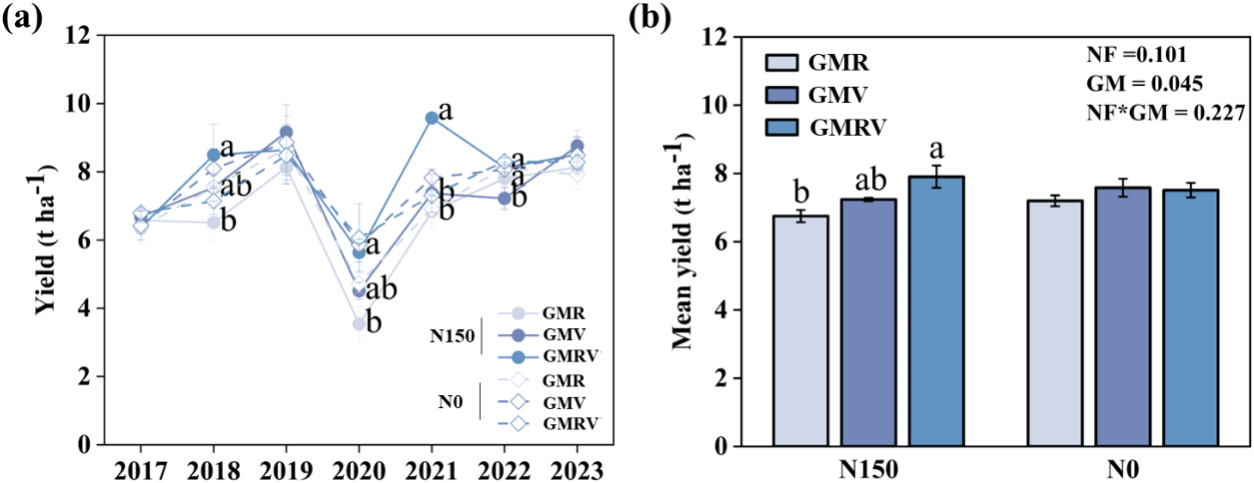
The wheat yield variation **(a)** and mean yield **(b)** depend on nitrogen fertilizer management (NF: N150, N0) and green manure types (GM: GMR, GMV and GMRV) from 2017 to 2023. GMR indicates with feed rape, GMV indicates with hairy vetch, GMRV indicates the combined application of non-leguminous and leguminous green manure. N150 and N0 represent nitrogen application rates of 150 and 0 kg ha^-1^, respectively. Values are means± standard errors (n = 3). Different lowercase letters indicate significant differences (p<0.05) among three green manure types within the same nitrogen fertilizer treatment.

**Figure 3:**
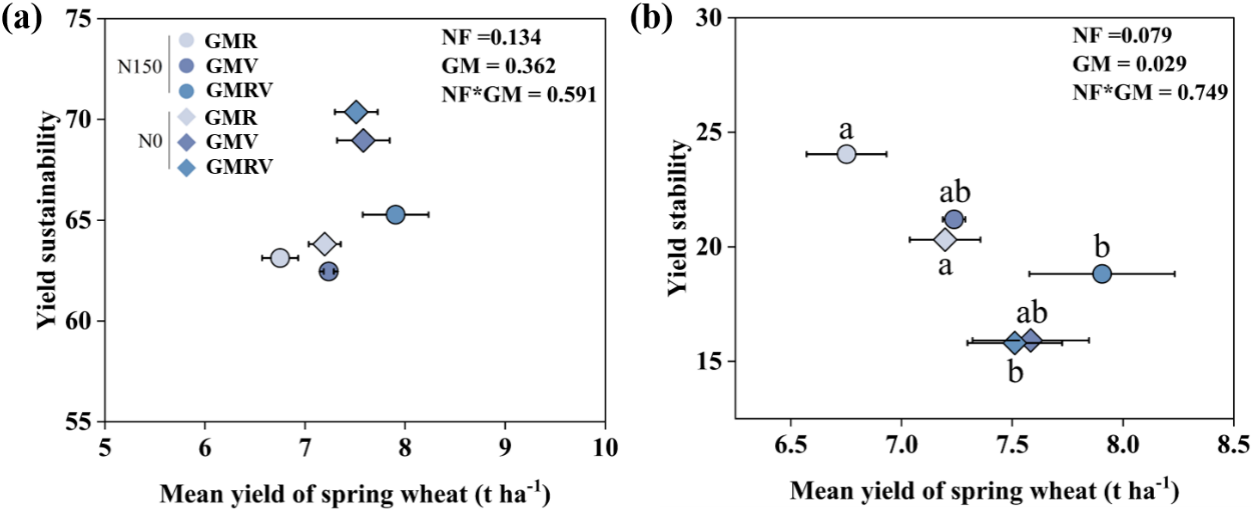
The sustainability **(a)** and yield stability **(b)** of spring wheat depend on nitrogen fertilizer management (NF: N150, N0) and green manure types (GM: GMR, GMV and GMRV) from 2017 to 2022. Values are means± standard errors (n = 3). GMR indicates with feed rape, GMV indicates with hairy vetch, GMRV indicates the combined application of non-leguminous and leguminous green manure. N150 and N0 represent nitrogen application rates of 150 and 0 kg ha^-1^, respectively. Different lowercase letters indicate significant differences (p<0.05) among three green manure types within the same nitrogen fertilizer treatment.

Spring wheat yield was significantly affected by both the experimental year and the type of green manure (*p* < 0.001) from 2017 to 2023 (**Supplementary Table S2**; **Figure 2**). The wheat yield in 2017–2023 were 6.54, 7.55, 8.66, 5.06, 7.64, 7.94 and 8.17 t ha^-1^, respectively. GMRV had significantly higher yield compared to GMR under N150 in 2018, 2020, 2021, and 2023, while both GMRV and GMR had significantly higher yield than GMV under N150 in 2022 (*p* < 0.05, **Figure 2**). The average wheat yield was 7.43 and 7.30 t ha^-1^ yr^-1^ under nitrogen fertilization and without nitrogen input. The average yield of GMR, GMV, GMRV under N150 were 6.8, 7.2 and 7.9 t ha^-1^ yr^-1^, and there was a significant difference between GMR and GMRV (*p* < 0.05, **Figure 2**). Further, GMRV enhanced subsequent spring wheat yield stability and sustainability under N150 and N0 (**Figure 3**). Green manure types had significant influence on yield stability (*p* < 0.05, **Figure 3b**). Relative to GMR, GMRV increased yield stability by 21.7% and 22.2% under N150 and N0, respectively (p< 0.05; **Figure 3b**). Overall, the combined incorporation of non-leguminous and leguminous green manure enhanced subsequent spring wheat yield while improving both yield stability and sustainability and reduced the dependence on nitrogen fertilization under green manure return conditions.

### 3.2 Yield components

**Figure 4:**
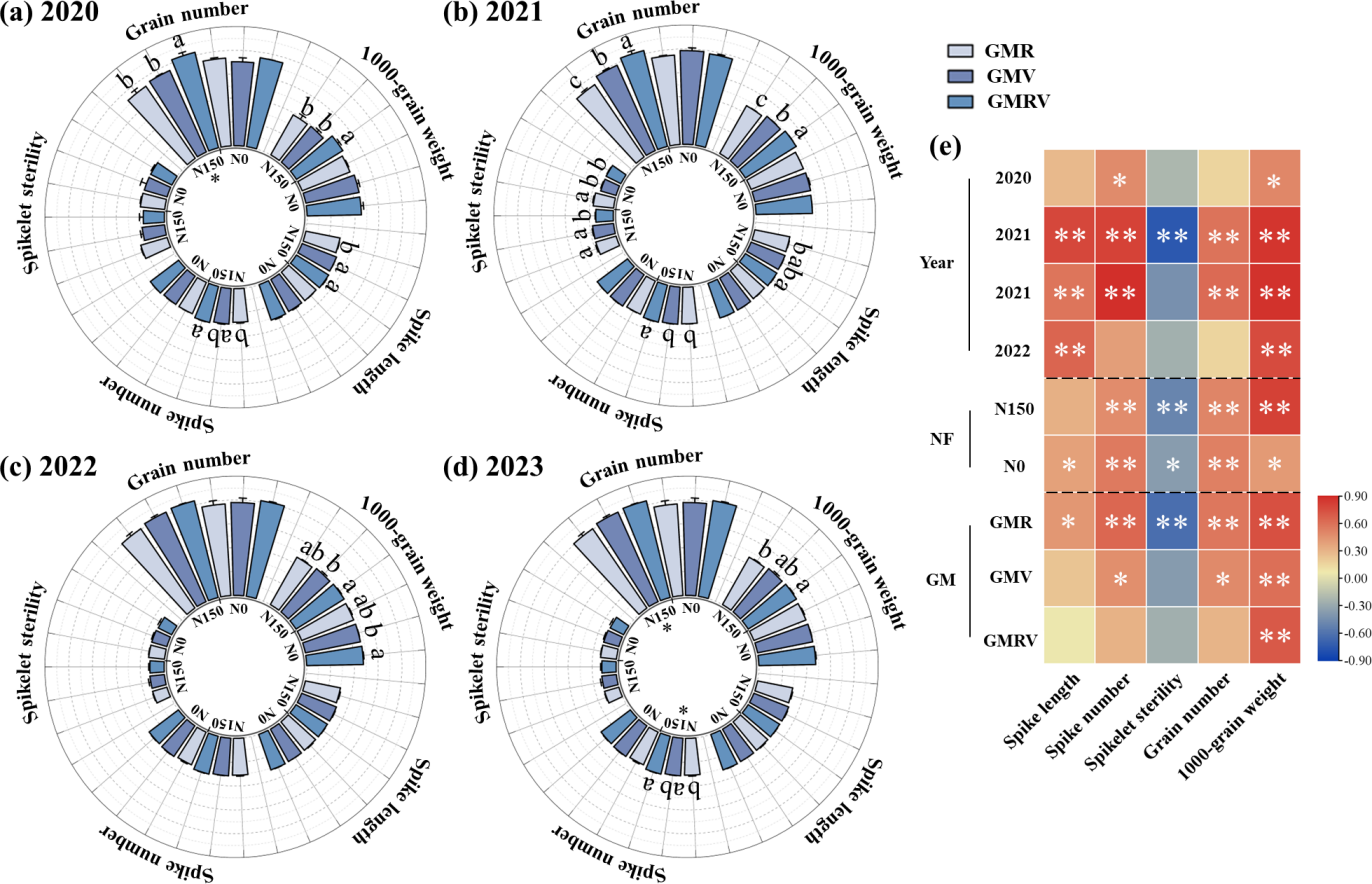
The wheat yield components on nitrogen fertilizer management (NF: N150, N0) and green manure types (GM: GMR, GMV and GMRV) in 2020 **(a)**, 2021 **(b)**, 2022 **(c)**, and 2023 **(D)**. Pearson correlation analysis between yield and yield components **(e)**. Values are means± standard errors (n = 3). GMR indicates with feed rape, GMV indicates with hairy vetch, GMRV indicates the combined application of non-leguminous and leguminous green manure. N150 and N0 represent nitrogen application rates of 150 and 0 kg ha-1, respectively. Different lowercase letters indicate significant differences (p<0.05) among three green manure types within the same nitrogen fertilizer treatment. *Indicates significant differences between two nitrogen fertilizer treatment at p< 0.05.

All yield components were significantly influenced by year (*p* < 0.001), and spike number, grain number and 1000-grain weight were also significantly influenced by green manure types from 2020-2023 (*p* < 0.01) (**Figure 4**). Spike length of GMRV significantly increased by 9.1% and 3.8% compared to GMV under N150 in 2020 and 2021, respectively (**Figures 4a, b**). Grain number of GMRV increased by 10.1%-12.7% and 5.5%-12.7% compared to GMV and GMR under N150 in 2020 and 2021, respectively (**Figures 4a, b**). Spike number of GMRV under N150 was higher than that of GMR except in 2022 (p< 0.05; **Figures 4a, b, d**). In addition, GMRV exhibited the highest 1000-grain weight from 2020 to 2023. Spike number and 1000-grain weight were positively correlated with yield across different years, nitrogen levels, and green manure treatments (p< 0.05; **Figure 4e**), indicating that these two traits were the primary yield components contributing to wheat yield.

### 3.3 Soil pH, EC, SOC, TN and TP

**Figure 5:**
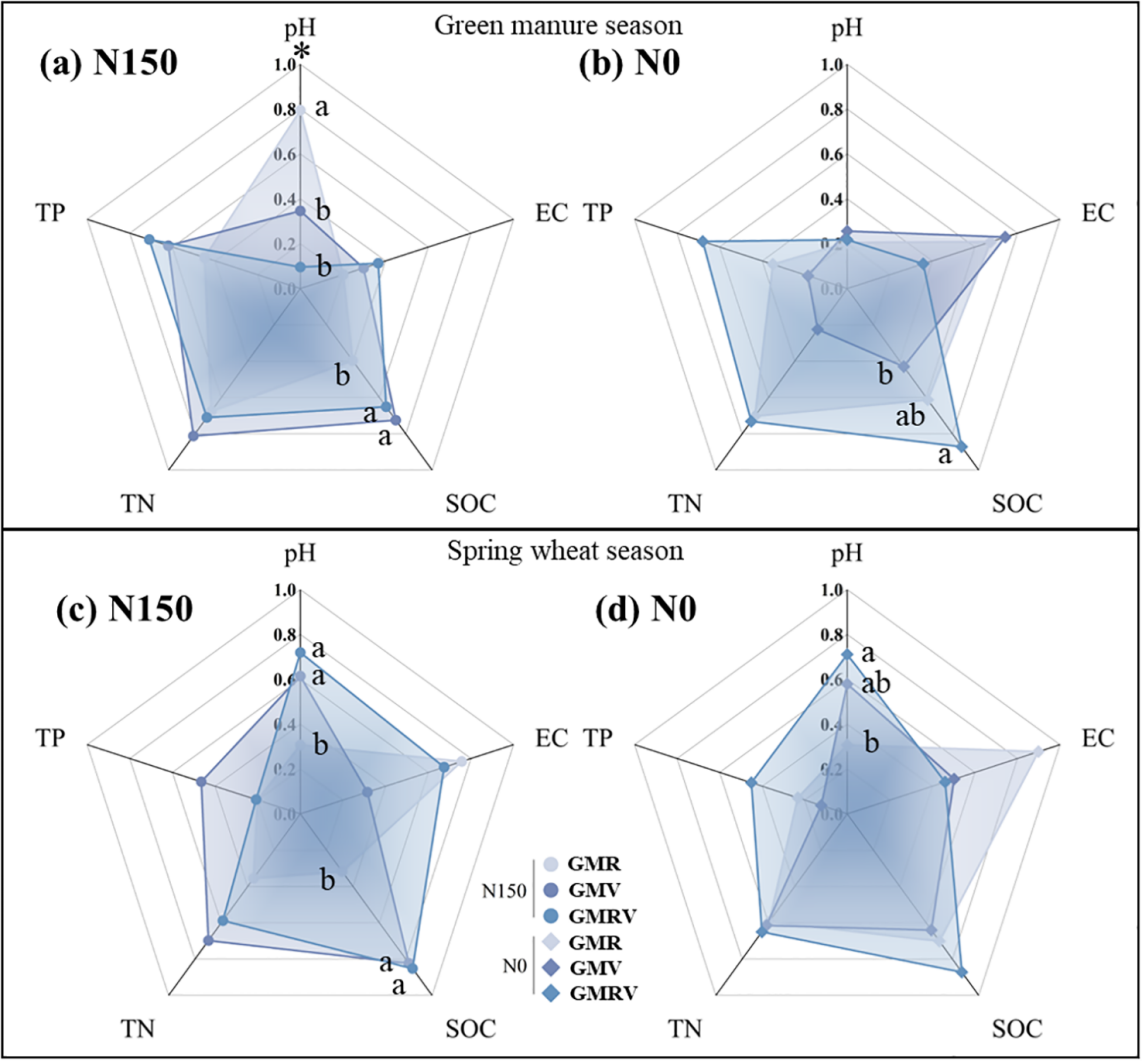
Correct version: Soil pH, SC, SOC, TN and TP at 0–20 cm soil layer depend on nitrogen fertilizer management (NF: N150, N0) and green manure types (GM: GMR, GMV and GMRV) in 2023. **(a)** Green manure season with N150; **(b)** green manure season with N0; **(c)** spring wheat season with N150; **(D)** spring wheat season with N0.GMR indicates with feed rape, GMV indicates with hairy vetch, GMRV indicates the combined application of nonleguminous and leguminous green manure. N150 and N0 represent nitrogen application rates of 150 and 0 kg ha^-1^, respectively. SC, salt content; SOC, soil organic carbon; TN, total nitrogen; TP, total phosphorus. Different lowercase letters indicate significant differences (p<0.05) among three green manure types within the same nitrogen fertilizer treatment.

At green manure season, the pH was 2.0% and 0.7% lower, and SOC were 13.6% and 10.5% higher under GMR and GMRV compared to GMV with N150, respectively (*p* < 0.05, **Figure 5a**). The SOC of GMRV improved by 14.5% compared to GMV with N0 (*p* < 0.05, **Figure 5b**). In spring wheat season, the green manure types had great influence on pH (**Figures 5c, d**). Compared to GMV, GMR and GMRV also significantly increased SOC by 6.3% and 6.7% with N150 (**Figure 5c**). In brief, GMRV exhibited higher SOC.

### 3.4 Soil enzyme activity and EMF

**Figure 6:**
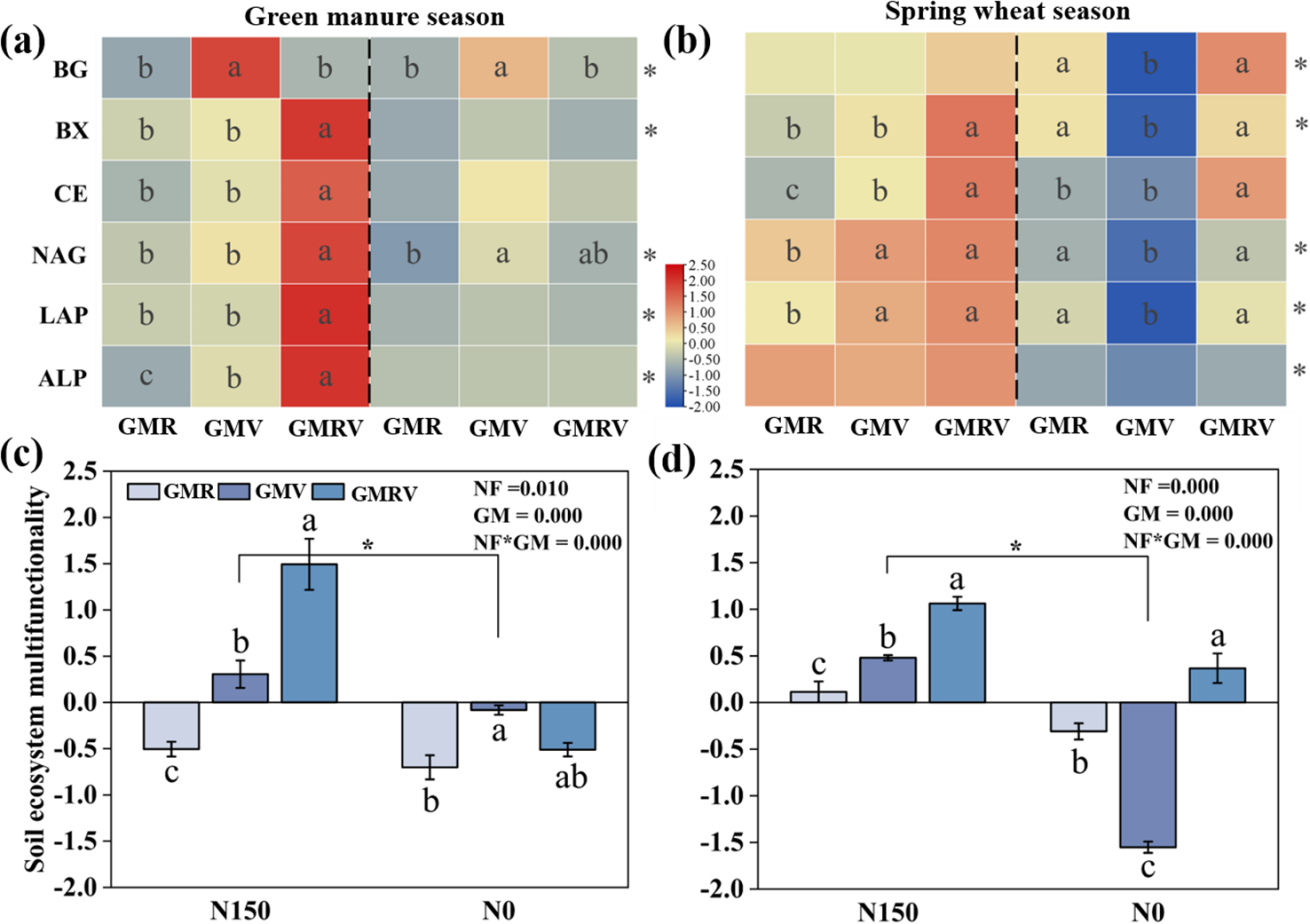
Heat map showing the changes in multiple enzyme activities of green manure season **(a)**and spring wheat season **(b)** across the 0–20 cm soil layer in 2023. The C-related enzymes include β-1, 4-glucosidase (BG), β-cellobiohydrolase (CBH), and β-xylosidase (BX); the N-related enzymes include β-1,4-Nacetylglucosaminidase (NAG) and L-leucine aminopeptidase (LAP), while P-related enzymes include alkaline phosphomonoesterase (ALP). The soil ecosystem multifunctionality of green manure season **(c)** and spring wheat season **(d)** was calculated based on the average standardized Z-score of six soil enzyme activities. GMR indicates with feed rape, GMV indicates with hairy vetch, GMRV indicates the combined application of non-leguminous and leguminous green manure. N150 and N0 represent nitrogen application rates of 150 and 0 kg ha^-1^, respectively. Different lowercase letters indicate significant differences (p<0.05) among three green manure types within the same nitrogen fertilizer treatment. * Indicates significant differences between two nitrogen fertilizer treatment at p< 0.05.

Soil enzyme activities were significantly influenced by green manure types and nitrogen fertilization, with higher values under N150 than N0 (p< 0.05; **Figures 6a, b**). During the green manure season, GMRV resulted in greater activities of BX, CE, LAP, NAG, and ALP, increasing by 123.8%–175.1%, 49.1%–81.4%, 62.9%–67.8%, 47.0%–76.1%, and 57.3%–92.1%, respectively, relative to GMR and GMV under N150 (p< 0.05; **Figure 6a**). During the spring wheat season, BX and CE activities under GMRV (N150) increased by 25.2%–41.1% and 24.7%–46.5%, respectively, compared with GMR and GMV (p< 0.05; **Figure 6b**). Similarly, LAP and NAG activities under GMV and GMRV (N150) were higher by 9.9%–14.8% and 12.0%–17.9%, respectively (p< 0.05; **Figure 6b**). Under N0, BG, BX, LAP, and NAG activities in GMR and GMRV increased by 71.0%–81.0%, 76.8%–81.5%, 37.9%–41.8%, and 52.0%–67.1%, respectively, relative to GMR (p< 0.05; **Figure 6b**).N fertilization, green manure type, and their interaction significantly affected EMF, with N150 showing higher values than N0 (p< 0.05; **Figures 6c, d**). GMRV exhibited significantly higher EMF than GMR and GMV under N150 in both the spring wheat and green manure seasons (p< 0.05; **Figures 6c, d**). Under N0, GMRV also maintained higher EMF than GMR and GMV during the spring wheat season. Overall, the combined incorporation of non-leguminous and leguminous green manure enhanced enzyme activities and improved EMF.

### 3.5 Soil enzymatic stoichiometry and microbial resource limitation

**Figure 7:**
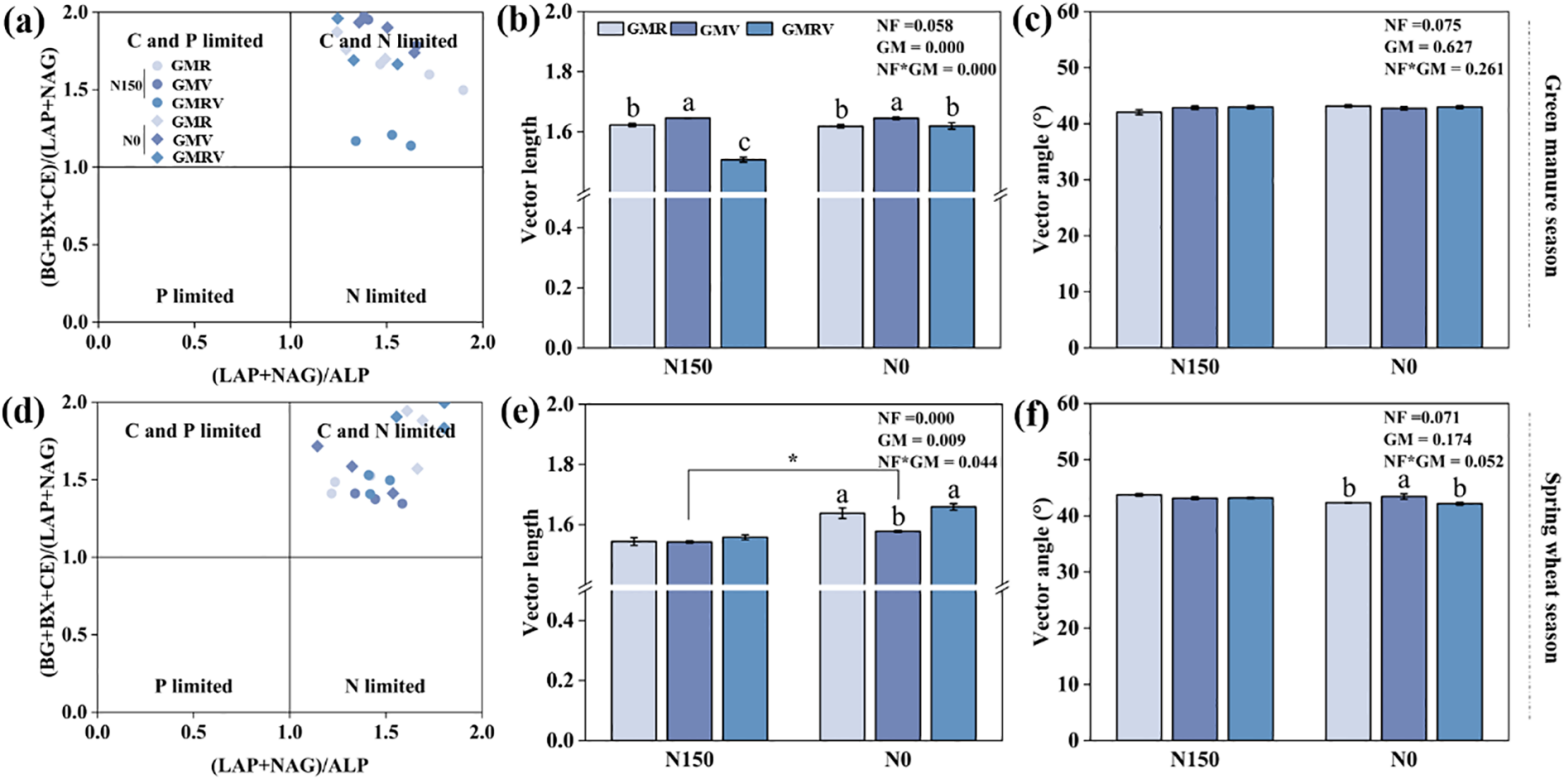
Soil scatter plot of soil enzymatic stoichiometry, soil enzyme vector length and vector angle of green manure season **(a–c)** and spring wheat season **(d–f)** at the 0–20 cm soil layer as affected by nitrogen fertilizer management (NF: N150, N0) and green manure types (GM: GMR, GMV and GMRV) in 2023. BG, β-1, 4-glucosidase; NAG, β-1,4-N-Acetyl-glucosaminidase; BX, β-xylosidase; CE, cellobiosidase; ALP, alkaline phosphatase; LAP, Leucine aminopeptidase. Different lowercase letters indicate significant differences (p< 0.05) among three green manure types within the same nitrogen fertilizer treatment.

The scatter plots of enzymatic stoichiometry showed that data points for GMR, GMV, and GMRV under both N150 and N0 were predominantly located within the C and N co-limitation region during both the green manure season (**Figure 7a**) and the spring wheat season (**Figure 7d**). Moreover, vector angles across all treatments were< 45°, which also indicated that microbial activity was limited by nitrogen resources (**Figures 7c, f**). During the green manure season, vector length under GMRV at both N150 and N0 was significantly lower than that under GMR and GMV, suggesting weaker microbial carbon (C) limitation (p< 0.05; **Figure 7b**). During the spring wheat season, vector length of GMR and GMRV under N0 significantly increased compared to GMV (p< 0.05; **Figure 7e**), while vector angle showed an opposite pattern (**Figure 7f**).

### 3.6 Correlation analysis

**Figure 8:**
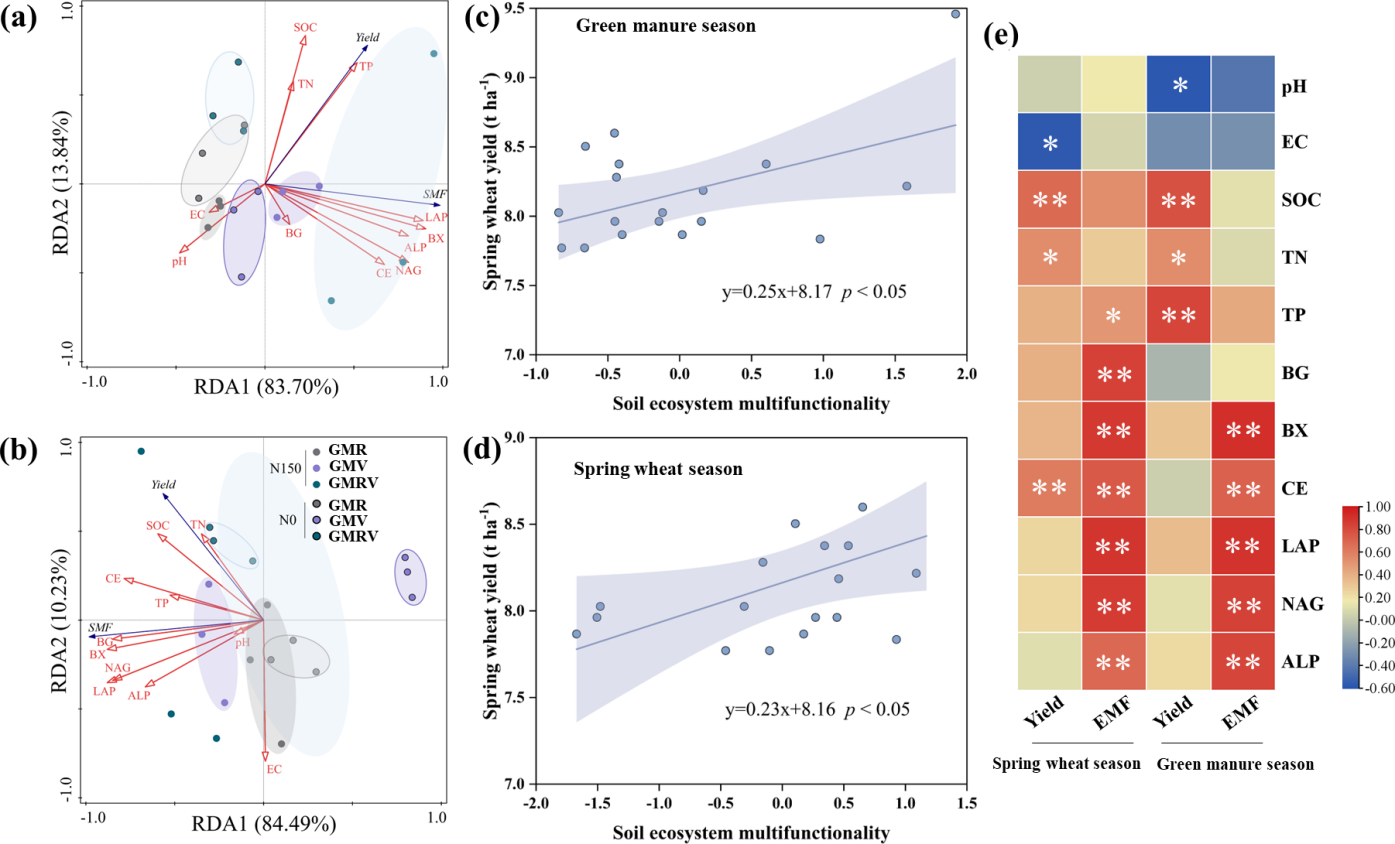
Redundancy analysis (RDA) of soil parameters, soil ecosystem multifunctionality (EMF) and spring wheat yield at green manure season **(a)** and spring wheat season **(b)**. Pearson correlation between the spring wheat yield and soil ecosystem multifunctionality at green manure season **(c)** and spring wheat season **(d)**. Heatmap of Pearson’s correlation coefficients between yield and soil parameters in spring wheat season and green manure season **(e)**. The shadow part represents the confidence interval. **p< 0.01, *p< 0.05.

Spring wheat yield showed significant positive correlations with SOC and TN, but was negatively associated with pH and EC (p< 0.05; **Figure 8e**). Soil ecosystem multifunctionality was positively correlated with enzyme activities and negatively associated with vector length (p< 0.05; **Figure 8e**). The study indicated a significant positive relationship between spring wheat yield and EMF (p< 0.05; **Figures 8c, d**). During the spring wheat season, soil parameters accounted for 97.8% of the variation in spring wheat yield and soil ecosystem multifunctionality (**Figure 8a**). Among these, BX, SOC, BG, and TP explained 69.3% (F = 36.1, p< 0.01), 15.9% (F = 16.2, p< 0.01), 3.6% (F = 4.6, p< 0.05), and 2.2% (F = 4.5, p< 0.05), respectively (**Figure 8a**). Similarly, during the spring wheat season, soil parameters explained 96.2% of the variation (**Figure 8b**). LAP was identified as the primary factor influencing spring wheat yield and soil ecosystem multifunctionality (F = 32.0, p< 0.01), accounting for 66.6% of the total variance, followed by SOC (12.6%, F = 9.2, p< 0.05) and BG (4.9%, F = 4.4, p< 0.05) (**Figure 8b**).

## 4 Discussion

### 4.1 Spring wheat yield response to green manure and nitrogen fertilizer

In the case of reducing N fertilizer, it is necessary to achieve stable yield through other measures. In this study, no significant differences in yield were observed between N150 and N0 under green manure treatments (**Figure 2**), indicating that green manure can partially compensate for reduced nitrogen application. As a clean biological N source, green manure provides a continuous supply of soil N, P, and other nutrients, thereby supporting wheat growth following long-term incorporation (Ma et al., 2021). Moreover, green manure return enhances soil physical structure and microbial properties, which facilitates root development and nutrient uptake (Yao et al., 2018). Overall, green manure plays a compensatory role in sustaining wheat yield under reduced nitrogen fertilization.

Our results further demonstrated that spring wheat yield, yield components, and yield stability were highest under GMRV (**Figures 2**, **3**), which is consistent with previous findings (Ma et al., 2021) showing that mixed sowing of leguminous and non-leguminous green manure produces higher yields than monoculture systems. This advantage can be attributed to differences in C/N ratios and decomposition dynamics between leguminous and cruciferous green manures (Pereirai et al., 2016). Hairy vetch, as a leguminous green manure, is characterized by a low C/N ratio and decomposes rapidly, releasing nitrogen (N) and accelerating soil N cycling, thereby promoting wheat tillering and nutrient uptake (Van der Pol et al., 2022; Xia et al., 2023). In contrast, feed rape, a cruciferous green manure with a higher C/N ratio, decomposes more slowly and provides a more prolonged residual effect. Through microbial decomposition processes, these residues contribute to soil organic matter formation (Pereirai et al., 2016), which enhances the availability of N and P and supports grain formation, thereby increasing grain number per spike and overall yield (Lu et al., 2007). These findings are further supported by our experimental results (**Figures 4**, **5**).

In this study, SOC under GMRV was higher (**Figure 6**), and SOC and TN showed significant positive correlations with wheat yield (**Figure 6f**). This relationship can be attributed to the decomposition of green manure, which releases substantial amounts of labile organic compounds and nutrients, including nitrogen (N), phosphorus (P), and potassium (K). Increased soil organic carbon alters soil physical structure and the microecological environment, thereby stimulating microbial activity and nutrient transformation processes (Yang et al., 2021). Soil C and N serve as key nutrient sources for plant growth, providing essential energy for yield formation (Liu et al., 2020; Gai et al., 2018). Furthermore, enhanced microbial activity and accelerated nutrient cycling driven by green manure residues promote greater nutrient uptake by wheat, ultimately leading to increased grain yield (Nunes et al., 2018; Garland et al., 2021; Li et al., 2023). The observed relationships between soil enzyme activities and wheat yield further support this mechanism (**Figures 2**, **3**, **6**). Consequently, the combined green manure represents an effective strategy for improving crop productivity, as it takes advantage of differences in decomposition rates and nutrient release patterns between leguminous and non-leguminous green manures to achieve complementary effects (Che et al., 2022), thereby supporting higher yields of subsequent spring wheat. Moreover, green manure incorporation provides a viable approach to reducing nitrogen fertilizer inputs.

### 4.2 Soil ecosystem multifunctionality and microbial resource limitation response to N fertilizer and green manure

Exogenous organic inputs provide essential substrates for microbial growth and can stimulate soil enzyme activities (He et al., 2021). In this study, GMRV increased soil enzyme activities, including BG, BX, CE, LAP, NAG, and ALP (**Figures 4a, b**), which is consistent with previous findings (Zhou et al., 2020). This effect may be explained by the root-mediated “puncturing effect” of feed rape, which enhances soil porosity and permeability, thereby improving soil physical structure. At the same time, green manure incorporation facilitates the mobilization and utilization of mineral phosphorus and promotes the accumulation of nutrients in surface soils after incorporation, which activates soil microorganisms and enhances enzyme activities (Duan et al., 2020; Jia et al., 2025; Khan et al., 2025; Yu et al., 2026);. In addition, biological nitrogen fixation by root nodules and the rapid decomposition of residues further contribute to increased enzyme activities in leguminous systems (Wang et al., 2023; Yang et al., 2024; Kama et al., 2025).Compared with N150, enzyme activities under N0 were reduced during both the spring wheat and green manure seasons (**Figures 4a, b**), which can be attributed to limited substrate availability under reduced nitrogen input (Wang et al., 2020). In contrast, N150 provides a greater supply of nitrogen to support green manure growth, thereby enhancing soil enzyme activities and plant development.

Our results indicate that green manure application enhanced soil EMF by promoting enzyme activities involved in C, N, and P cycling (**Figure 8e**). Specifically, GMRV consistently exhibited higher EMF than GMR and GMV during both the green manure and spring wheat seasons. Similarly, Liu et al. (2025) also indicated that green manure cultivation induced significantly higher EMF, and these improvements were associated with enhanced functioning of nutrient-cycling enzymes (Liu et al., 2025). Furthermore, a significant positive relationship between spring wheat yield and EMF was observed in this study (**Figure 5c**). The combined application of green manure not only supplied nutrients (**Figure 5**) and stimulated microbial activity (**Figures 6a, b**), but also enhanced multiple soil ecosystem functions, including carbon sequestration (Liu et al., 2025; Yu et al., 2024). These improvements in soil ecosystem multifunctionality contributed to increased crop productivity. Ultimately, the combined incorporation of non-leguminous and leguminous enhances enzyme activities and optimizes soil ecosystem multifunctionality, thereby creating a favorable soil microenvironment and improving subsequent spring wheat yield within the wheat–green manure cropping system.

Microbial C and N limitations were observed under all treatments whether it is wheat season or green manure season (**Figures 5**, **6**). In the Hetao Irrigation District, long-term irrigation with Yellow River water has contributed to severe secondary soil salinization (Lei et al., 2001). This process is exacerbated by low precipitation and poor soil permeability, resulting in reduced soil organic matter and enzyme activities (Song et al., 2022). These conditions collectively contribute to pronounced microbial C and N limitations in this region. In addition, lower vector length means lower microbial C limitation (Moorhead et al., 2013), GMRV had lower microbial C limitation under N150 and N0 at green manure season (**Figure 7b**), indicating that soil C limitation was alleviated. When leguminous and non-leguminous green manure is combined, complementary spatial distribution between above ground biomass and root systems allows for more efficient utilization of carbon resources. Higher green manure biomass also supports this view (**Supplementary Figure 1**). At spring wheat season, we observed the opposite results. GMRV under N0 had higher microbial C limitation at spring wheat season. The substantial labile carbon input from green manure incorporation is rapidly consumed by microorganisms during the early growth stage of spring wheat. In the later growth stage of spring wheat, the remaining green manure residues are predominantly composed of recalcitrant carbon components, while the microbial biomass stimulated by green manure still maintains a high demand for labile carbon, thereby exacerbating microbial carbon limitation (Wang et al., 2022; Song et al., 2023). Increased carbon acquisition activity further elevates carbon demand, exacerbating microbial C limitation (**Figure 6b**). Notably, nitrogen fertilization significantly reduced microbial C limitation during the spring wheat season (**Figure 7e**), indicating that the absence of external nitrogen inputs may intensify carbon limitation for microbial communities (Ren et al., 2016; Cui et al., 2022). Moreover, the negative relationship between vector length and EMF suggests that alleviating microbial C limitation contributes to improved soil ecosystem multifunctionality (**Figure 8e**; Chu et al., 2024). Overall, the combined incorporation of non-leguminous and leguminous enhanced nutrient availability and bioavailability in soil, thereby improving soil ecosystem multifunctionality and alleviating microbial carbon limitation, ultimately supporting the sustainability of ecosystem services.

## 5 Conclusions

A nine-year long-term field study confirmed that reducing nitrogen fertilizer application did not decrease spring wheat yield. The co-application of non-leguminous feed rape and leguminous hairy vetch green manure enhanced subsequent spring wheat yield, yield components and yield stability, and had higher SOC, enzyme activities and soil ecosystem multifunctionality. Spring wheat yield was positively associated with SOC, TN, and EMF. Co-incorporating non-leguminous feed rape and leguminous hairy vetch green manure alleviated the microbial carbon limitation in the green manure season, while leguminous green manure alleviated the microbial carbon limitation in the spring wheat season. Overall, this study suggests that co-application of non-leguminous and leguminous green manure is beneficial for enhancing spring wheat yield, improving soil ecosystem multifunctionality, and alleviating microbial carbon limitation within a wheat-green manure cropping system in saline soils.

## Data Availability

The raw data supporting the conclusions of this article will be made available by the authors, without undue reservation.
