# Supplementary material for: Co-application of leguminous and non-leguminous green manures enhances subsequent wheat yield stability in saline-alkali soils
Source: Front Plant Sci. 2026 Jun 17;17:1850670. doi: 10.3389/fpls.2026.1850670 (PMC13318794; doi:10.3389/fpls.2026.1850670)
Supplement: Supplementary file 1 [file Supplementaryfile1.docx]

**Table S1** Fertilization rate of experimental plot

| Nitrogen fertilizer  (NF) | Green manure  (GM) | Basic fertilizer  (kg ha^-1^） | | | | Additional fertilizer  (kg ha^-1^） |
| --- | --- | --- | --- | --- | --- | --- |
|  |  | urea  N: 46% | diammonium phosphate  N: 18%  P_2_O_5_: 46% | Superphosphorus  P_2_O_5_: 46% | potassium chloride  K_2_O: 95% | urea  N: 46% |
| N | R | 85 | 195 | - | 80 | 165 |
|  | V | 85 | 195 | - | 80 | 165 |
|  | RV | 85 | 195 | - | 80 | 165 |
| N0 | R | - | - | 195 | 80 | - |
|  | V | - | - | 195 | 80 | - |
|  | RV | - | - | 195 | 80 | - |

**Table S2** Combined analysis of variances for spring wheat yield in 2017-2023 and agronomical and physiological

traits in 2020-2023.

| Traits | Source of variation | | | | | | |
| --- | --- | --- | --- | --- | --- | --- | --- |
|  | Year  （Y） | Nitrogen fertilizer  (NF) | Green manure  (GM) | Y×NF | Y×GM | NF×GM | Y×NF×GM |
| Wheat yield | *** | ns | *** | ns | ns | ns | ns |
| Spike length | * | ns | ns | ns | ns | ns | ns |
| Spike number | *** | ns | ** | ns | ns | * | ns |
| Spikelet sterility | *** | ns | ns | ns | ns | ns | ns |
| Grain number | ** | ** | ** | ns | ns | ns | ns |
| 1000-grain weight | ** | ns | *** | * | ns | ns | ns |


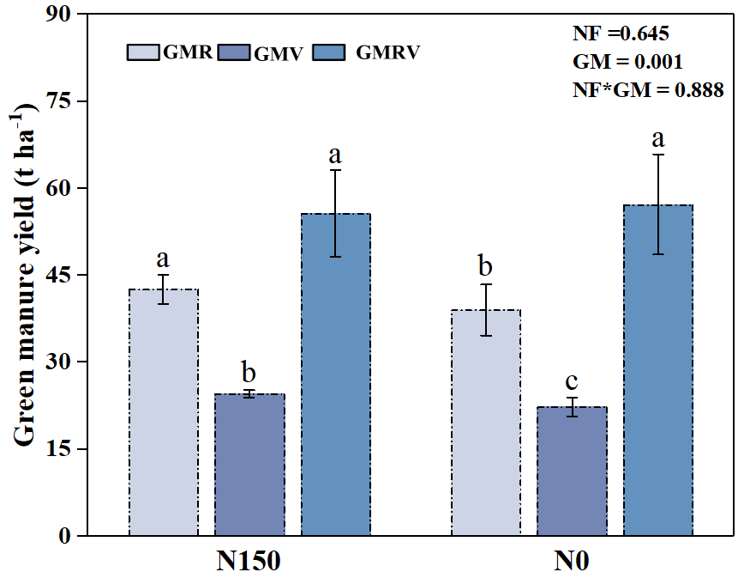


**Fig. S1.** Green manure yield in 2022.
